# Sex Hormones Regulate Tenofovir-Diphosphate in Female Reproductive Tract Cells in Culture

**DOI:** 10.1371/journal.pone.0100863

**Published:** 2014-06-30

**Authors:** Zheng Shen, John V. Fahey, Jack E. Bodwell, Marta Rodriguez-Garcia, Angela D. M. Kashuba, Charles R. Wira

**Affiliations:** 1 Department of Physiology and Neurobiology, Geisel School of Medicine at Dartmouth, Lebanon, New Hampshire, United States of America; 2 Division of Pharmacotherapy and Experimental Therapeutics, University of North Carolina Eshelman School of Pharmacy, Chapel Hill, North Carolina, United States of America; Temple University School of Medicine, United States of America

## Abstract

The conflicting results of recent pre-exposure prophylaxis (PrEP) trials utilizing tenofovir (TFV) to prevent HIV infection in women led us to evaluate the accumulation of intracellular TFV-diphosphate (TFV-DP) in cells from the female reproductive tract (FRT) and whether sex hormones influence the presence of TFV-DP in these cells. Following incubation with TFV, isolated epithelial cells, fibroblasts, CD4^+^ T cells and CD14^+^ cells from the FRT as well as blood CD4^+^ T cells and monocyte-derived macrophages convert TFV to TFV-DP. Unexpectedly, we found that TFV-DP concentrations (fmol/million cells) vary significantly with the cell type analyzed and the site within the FRT. Epithelial cells had 5-fold higher TFV-DP concentrations than fibroblasts; endometrial epithelial cells had higher TFV-DP concentrations than cells from the ectocervix. Epithelial cells had 125-fold higher TFV-DP concentrations than FRT CD4^+^ T cells, which were comparable to that measured in peripheral blood CD4^+^ T cells. These findings suggest the existence of a TFV-DP gradient in the FRT where epithelial cells > fibroblasts > CD4^+^ T cells and macrophages. In other studies, estradiol increased TFV-DP concentrations in endometrial and endocervical/ectocervical epithelial cells, but had no effect on fibroblasts or CD4^+^ T cells from FRT tissues. In contrast, progesterone alone and in combination with estradiol decreased TFV-DP concentrations in FRT CD4^+^ T cells. Our results suggest that epithelial cells and fibroblasts are a repository of TFV-DP that is under hormonal control. These cells might act either as a sink to decrease TFV availability to CD4^+^ T cells and macrophages in the FRT, or upon conversion of TFV-DP to TFV increase TFV availability to HIV-target cells. In summary, these results indicate that intracellular TFV-DP varies with cell type and location in the FRT and demonstrate that estradiol and/or progesterone regulate the intracellular concentrations of TFV-DP in FRT epithelial cells and CD4^+^ T cells.

## Introduction

The Human Immunodeficiency Virus (HIV) global pandemic has become one of the world's most serious health challenges. There were 35.3 million people living with HIV at the end of 2012 and about 2.3 million new infections during 2012 [1]. Worldwide, the majority of new cases are spread by vaginal and anal sexual intercourse, with a higher proportion of women infected via heterosexual intercourse than men [2]. Younger age, sexual violence, and co-infection with sexually transmitted infections (STI) are among the risk factors that contribute to susceptibility to HIV infection [3], [4].

The female reproductive tract (FRT) is the primary mucosal site of infection by STDs including HIV. Unique among mucosa sites, the FRT is exposed to large fluxes in the levels of the sex hormones estradiol (E_2_) and progesterone (P4) across the menstrual cycle, and at concentrations higher than those observed elsewhere in the body. Sex hormone modulation of innate and adaptive immune protection led to the hypothesis of a “Window of Vulnerability” occurring during the later half of the menstrual cycle, when HIV and other sexually transmitted pathogens are most likely to infect women [5]. The FRT mucosa is composed of multiple cell types including epithelial cells, fibroblasts and immune cells. Each plays a central role in providing cellular, humoral, and innate immune protection against bacterial and viral invasion as well as physiological changes for reproductive success [6], [7].

Recently, Pre-exposure Prophylaxis (PrEP) studies with anti-retroviral drugs to prevent infection has provided hope to reduce the dimensions of the HIV pandemic. For example, the nucleoside-analog reverse transcriptase inhibitor (NRTI) tenofovir demonstrated efficacy in in vitro studies, animal models and initial clinical trials [8], [9]. However, the use of oral TFV and TFV as a vaginal gel in the Vaginal and Oral Interventions to Control the Epidemic (VOICE) trial [10] failed to protect women against the sexual acquisition of HIV [11], [12].

Benefits of TFV include suppression of viral replication, a favorable safety profile and a relatively long half-life [13]. After entering the cell, TFV requires two phosphorylation steps to be activated into TFV-diphosphate (TFV-DP) [14]. TFV-DP can compete for dATP during the HIV reverse transcriptase step and, once incorporated into the nascent viral cDNA, causes chain termination and thus inhibits viral replication. Since microbicides are administered vaginally in gels or taken orally, it is important to measure intracellular concentrations to be certain that TFV has been absorbed in the mucosal tissue. TFV and TFV-DP concentrations in human plasma, PBMCs, as well as genital and colorectal tissues, have been measured for a number of HIV prevention studies [11]. These studies have suggested a correlation between drug concentration and efficacy. However, the intracellular concentration of TFV-DP within individual cell types (epithelial cells, fibroblasts and immune cells) at different sites (endometrium (EM), endocervix (CX), ectocervix (ECX)) in the FRT has not been studied. Additionally, the effects of sex hormones and chemical contraceptives on TFV-DP concentrations are poorly understood.

In this study, we evaluated the effects of E_2_ and/or P4 on TFV-DP concentrations (fmol/million cells) in epithelial cells, fibroblasts and immune cells (CD4^+^ T cells and macrophages) from sites in the upper (EM, CX) and lower (ECX) FRT. The rationale for this study was based on the recognition that to reach HIV-target cells embedded in the stroma of the FRT, TFV taken orally must permeate endothelial cells to enter the FRT stroma, or in the case of vaginal deposition, move through and/or between epithelial cells and fibroblasts to reach HIV-target cells (CD4^+^ T cells, macrophages and dendritic cells) in the upper and lower FRT. The goals of this study were to evaluate the ability of isolated cells to activate TFV and to determine whether E_2_ and P4 regulate the TFV-DP concentrations in epithelial cells, fibroblasts and immune cells throughout the FRT.

## Materials and Methods

### Source of tissue and blood

Human FRT tissues were obtained immediately following surgery from women who had undergone hysterectomies at Dartmouth-Hitchcock Medical Center (Lebanon, NH). Tissues from the EM, CX, and ECX were collected from hysterectomy patients with benign conditions such as fibroids and prolapse (age from 41 to 66). Tissue samples were distal from the sites of pathology and were without pathological lesions as determined by a pathologist. Blood donors were anonymous, no information regarding age or hormonal status was available and only female donors were used in this study. All human subject work was carried out with the approval of the Dartmouth College Institutional review Board. Approval to use tissues was previously obtained from the Committee for the Protection of Human Subjects (CPHS), and with written informed consent obtained from the patient before surgery.

### Tissue processing

Tissues were rinsed with 1x HBSS (Hanks balanced salt solution) supplemented with phenol red, 100 U/ml penicillin, 100 µg/ml streptomycin (all Thermo Scientific Hyclone, Logan, UT), and 0.35 mg/ml NaCO_3_ (Fisher Scientific, Pittsburgh, PA). Tissues were then minced under sterile conditions into 1–2 mm fragments and digested at 37°C for 1 hr using a mixture containing (final concentrations): 0.05% collagenase type IV (Sigma-Aldrich, St. Louis, MO) and 0.01% DNAse (Worthington Biochemical, Lakewood, NJ) in 1xHBSS (Invitrogen Life Technologies, Grand Island, NY). Type IV collagenase was selected based on preliminary studies to ensure non-cleavage of surface markers (Rodriguez-Garcia et al, submitted). After digestion, cells were dispersed through a 250-µm nylon mesh screen (Small Parts, Miami Lakes, FL), washed, and resuspended in complete media consisting of DMEM/F12 medium without phenol red, supplemented with 10 µM HEPES (both GIBCO, Life Technologies, Grand Island, NY), 100 µg/ml primocin (InvivoGen, San Diego, CA), 2 mM L-glutamine, 2.5% heat-inactivated defined fetal Bovine Serum (FBS) (both from Thermo Scientific Hyclone) and 2.5% NuSerum (BD Biosciences, Bedford, MA). Epithelial cell sheets were separated from stromal cells by filtration through a 20-µm mesh filter (Small Parts). Epithelial cell sheets were retained on the filter, while stromal cells passed through. Stromal cells were then washed and counted and dead cells removed using the Dead cell removal kit (Miltenyi Biotec, Auburn, CA) according to manufacturer instructions to obtain a mixed cell suspension for isolation of CD14^+^ cells, CD4^+^ T cells and fibroblasts.

### Isolation and culture of FRT epithelial cells

Epithelial cell sheets were recovered by rinsing and backwashing the filter with complete medium, centrifuged at 500×g for 5 min and analyzed for cell number and viability. To establish a cell culture system of polarized human FRT epithelial cells with both apical and basolateral compartments, FRT epithelial cells were cultured in Matrigel matrix (BD Biosciences) coated Falcon cell culture inserts in 24-well companion culture plates (Fisher Scientific). Apical and basolateral compartments contained 300 and 500 µl of complete medium, respectively, which was changed every 2 days. Tight junction formation of epithelial cell monolayers from EM and CX was assessed by periodically measuring transepithelial resistance (TER) using an EVOM electrode and Voltohmmeter (World Precision Instruments, Sarasota, FL), as described previously [15]–[17]. To keep the culture conditions similar, the same procedure was followed for culturing squamous ECX epithelial cells, which do not polarize.

### Isolation and culture of FRT CD14^+^ cells

Following removal of dead cells, CD14^+^ cells were isolated using positive magnetic bead selection with the CD14 MicroBeads (Miltenyi Biotec) according to the manufacturer's instructions. After two rounds of positive selection, purity of the CD14^+^ cell population was higher than 90% (Rodriguez-Garcia et al, submitted). Freshly isolated CD14^+^ cells were plated at a density of 1×10^5^ cells per well in ultra-low attachment 96-well culture plate (Corning, Corning, NY) in 0.2 ml of immune cell media consisting of X-VIVO 15 Media (Lonza, Walkersville,MD) supplemented with 10% charcoal stripped human AB serum (Valley Biomedical, Winchester, VA) prior to treatment.

### Isolation and culture of FRT CD4^+^ T cells and fibroblasts

Following removal of CD14^+^ cells, CD4^+^ T cells were isolated from each cell suspension by negative magnetic bead selection with the CD4^+^ T cell isolation kit (Miltenyi Biotec) following instructions with minor modifications, as previously described (Rodriguez-Garcia et al, submitted). Anti-fibroblast microbeads (Miltenyi Biotec) were added in combination with the microbeads supplied with the kit to ensure depletion of stromal fibroblasts present in the mixed cell suspension. After two rounds of negative selection, purity of the CD4^+^ T cell population was higher than 90% (Rodriguez-Garcia et al, submitted). Freshly isolated CD4^+^ T cells from tissues were plated at a density of 1×10^5^ cells per well in an ultra-low attachment 96-well culture plate (Corning, Corning, NY) in 0.2 ml of immune cell media prior to treatment.

Fibroblasts were collected by positive selection using anti-fibroblast microbeads as described above to purify CD4^+^ T cells. Fibroblasts were cultured in T75 flasks (Falcon, Fisher Scientific, Pittsburgh, PA) in complete medium as described above. The medium was changed every 2 days for 4-6 days to remove non-adherent cells. Purity was verified by intracellular staining of vimentin and surface expression of CD90 and lack of CD45 [18], [19]. Once fibroblasts reached confluence, they were trypsinized with 0.05% trypsin-EDTA (GIBCO, Life Technologies) and seeded into an ultra-low attachment 24-well culture plate (Corning, Corning, NY) at a density of 1×10^5^ cells per well (unless otherwise indicated) in 500 µl complete medium prior to treatment, as described previously [20].

### Preparation of blood Macrophages and CD4^+^ T cells

Peripheral blood mononuclear cells (PBMC) were isolated by standard Ficoll density gradient centrifugation. To generate monocyte-derived macrophages, as described previously [21], CD14^+^ cells were isolated using positive magnetic bead selection with the CD14 MicroBeads (Miltenyi Biotec) following instructions. Purity higher than 98% was obtained for CD14^+^ cell populations after magnetic isolation by using Flow cytometry analysis (data not shown). CD14^+^ cells were cultured in ultra-low attachment T25 flasks (Corning, Corning, NY) with immune cell media as described above. After 4 days, macrophages were plated at a density of 1×10^5^ cells per well in ultra-low attachment 96-well culture plate in 0.2 ml of immune cell media.

Blood CD4^+^ T cells were purified from PBMC using negative magnetic bead selection with the CD4^+^ T cell isolation kit (Miltenyi Biotec). Purity higher than 98% was obtained for CD4^+^ T cell populations after magnetic isolation by using Flow cytometry analysis (data not shown). Freshly isolated CD4^+^ T cells were plated at a density of 1×10^5^ cells per well (unless otherwise indicated) in an ultra-low attachment 96-well culture plate in 0.2 ml of immune cell media.

### TFV preparation

TFV in powder form was obtained from AIDS Research and Reference Reagent Program (NIH AIDS Reagent Program, Division of AIDS, NIAID, NIH: Tenofovir, catalog number 10199). A stock concentration of TFV 10 mg/ml was prepared by adding 1 ml of PBS to 10 mg of TFV powder. A final concentration of 1 mg/ml of TFV was used unless otherwise indicated.

### Hormone preparation

17β-estradiol (Calbiochem, Gibbstown, NJ) and progesterone (Calbiochem) was dissolved in 100% ethanol for an initial concentration of 1×10^−3^ M, evaporated to dryness and suspended in media containing 10% charcoal dextran-stripped FBS or immune cell complete media to a concentration of 1×10^−5^ M. Further dilutions were made to achieve final working concentration, and cells were treated with 5×10^−8^ M E_2_ and/or 1×10^−7^ M P4. Both are standard hormone treatment concentrations used by our laboratory and each is within the physiological range of hormone concentration [22]. As a control, an equivalent amount of ethanol without dissolved hormone was initially evaporated.

### TFV and hormone treatment

Epithelial cells and fibroblasts in culture were switched to complete media containing 10% of charcoal dextran-stripped FBS prior to TFV and hormone treatment. After 24 hr, the media was replaced and cells were treated with TFV and hormone for 24 hr. Fresh isolated CD4^+^ T cells and CD14^+^ cells both from tissues and blood were treated with TFV and hormone for 24 hr. Hormone or ethanol control media was added to both the apical and basolateral compartments (epithelial cells) or to individual wells for all other cells. TFV was added to apical compartment only for epithelial cell cultures, and to individual wells for all other cells. After 24 hr treatment, cells were harvested and lysed in 300 µl of 70% methanol, and stored immediately at −80°C prior to TFV-DP evaluation as previously described [23]. Intracellular TFV-DP concentrations were measured by liquid chromatography with tandem mass spectrometry (LC-MS/MS) and normalized for cell count [23].

### Statistics

Data analysis was performed using the GraphPad Prism 5.0 (GraphPad Software, San Diego, CA). A two sided P value<0.05 was considered statistically significant. Comparison of three or more groups was performed applying One-way ANOVA with Bonferroni post test. Comparison of normalized data was performed applying column statistics – one sample t test, which compares the mean of every column of data to the hypothetical value (controls are by definition all equal to 100).

## Results

### TFV-DP is produced in multiple cell types

**Figure 1:**
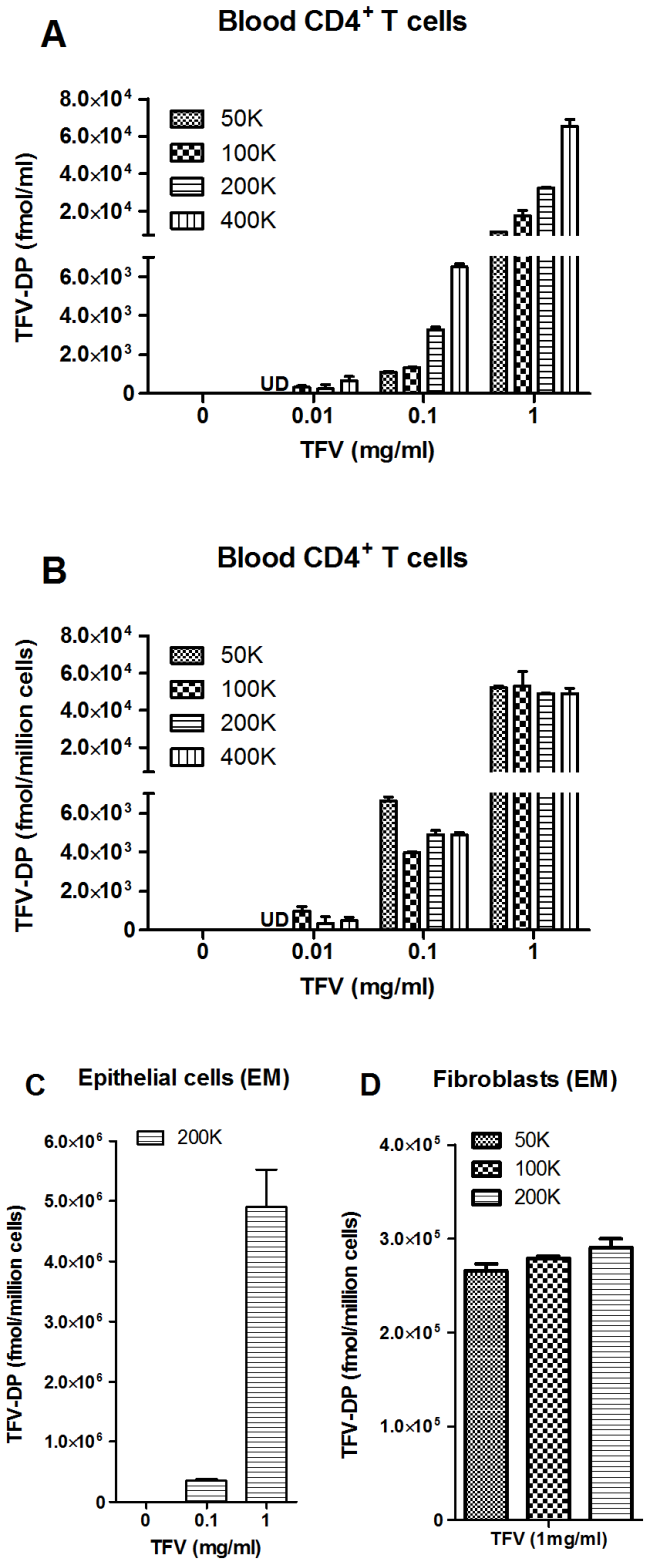
Blood CD4^+^ T cells and endometrial epithelial cells and fibroblasts metabolize TFV to TFV-DP. TFV-DP levels were measured by LC-MS/MS in increasing numbers of purified blood CD4^+^ T cells (A and B) and EM epithelial cells (C) and fibroblasts (D) treated with indicated dose of TFV for 24 hr. Bars represent mean and SEM from triplicate cultures. Values are expressed as fmol per ml in (A) and fmol/million cells in (B, C and D).

To obtain direct evidence on the extent to which cells are able to activate TFV, we isolated blood CD4^+^ T cells and monocyte-derived macrophages as well as endometrial fibroblasts and epithelial cells and analyzed them for their ability to convert TFV to TFV-DP. To optimize conditions for measuring TFV-DP, increasing numbers of purified blood CD4^+^ T cells were incubated with TFV (0.01–1.0 mg/ml) for 24 hr prior to measuring TFV-DP concentrations by LC-MS/MS. As seen in **Figure 1A**, we found that treating 50–400 K cells with TFV at 0.1 and 1 mg/ml resulted in measurable TFV-DP. Lower concentrations of TFV (0.01 mg/ml) at the cell numbers analyzed were insufficient to measure TFV-DP. To optimize assay conditions, we analyzed the data for blood CD4^+^ T cells on TFV-DP concentrations (fmol/million cells) in **Figure 1A**. As shown in **Figure 1B**, we obtained comparable TFV-DP over a range of cell concentrations when adjusting for cell number (fmol/million cells). Based on these analyses, we concluded that 100 K CD4^+^ T cells and 1 mg/ml TFV were optimal for cell number and TFV concentration, respectively. In other studies (not shown), we found that 100 K cells and 1 mg/ml TFV was optimal for measuring TFV-DP in blood monocyte-derived macrophages.

To determine if EM epithelial cells and fibroblasts metabolize TFV to form TFV-DP, isolated cells were incubated with TFV for 24 hr prior to measuring TFV-DP. As seen in **Figure 1C**, we found that TFV-DP increased with a higher dose of TFV in EM epithelial cells (200 K). When we increased the number of EM fibroblasts (50–200 K), we found higher concentrations of TFV-DP with optimal 1 mg/ml of TFV (data not shown). When we analyzed TFV-DP by fmol/million cells (**Figure 1D**), we determined that 100 K were adequate for studies with FRT fibroblasts. To the best of our knowledge, this is the first demonstration that TFV-DP is formed in epithelial cells and fibroblasts from the FRT. We also measured TFV-DP in CD4^+^ T cells and CD14^+^ cells from FRT tissues and found that treating 100 K cells with TFV at 1 mg/ml resulted in measurable TFV-DP (data not shown).

### 2. TFV-DP within FRT tissues vary with the individual cell types analyzed

**Figure 2:**
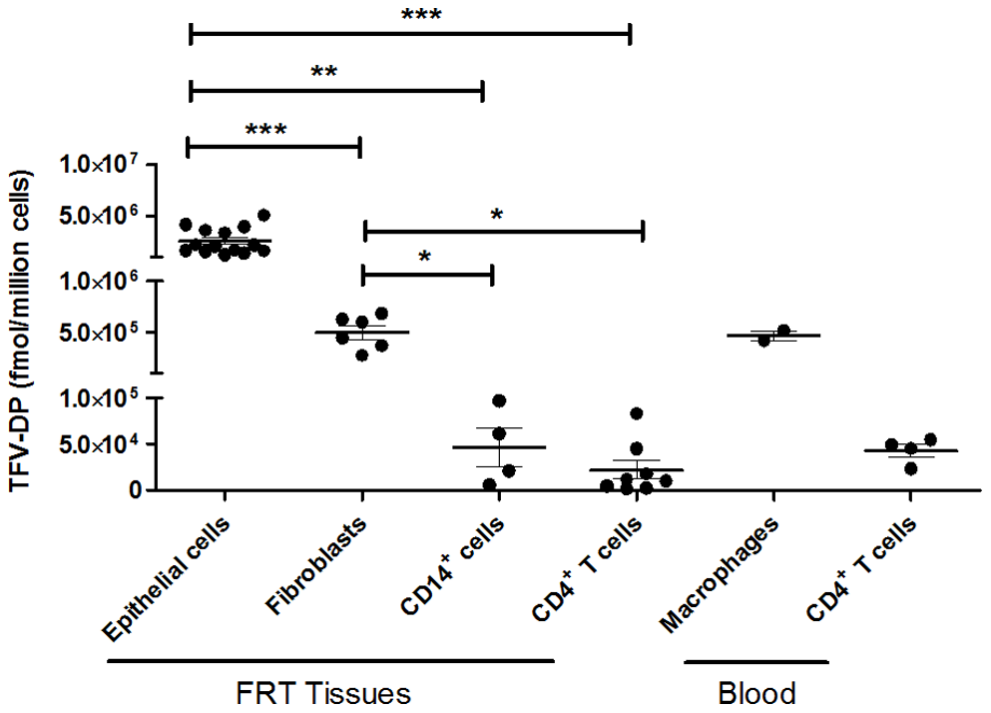
Comparison of TFV-DP levels in epithelial cells, fibroblasts, CD14^+^ cells and CD4^+^ T cells from FRT tissues. TFV-DP levels were measured by LC-MS/MS in purified cultures of epithelial cells (n = 14), fibroblasts (n = 6), CD14^+^ cells (n = 4) and CD4^+^ T cells (n = 8) from FRT tissues as well as monocyte-derived macrophages (n = 2) and CD4^+^ T cells (n = 4) from blood treated with TFV (1 mg/ml) for 24 hr. Each circle represents FRT cells from an individual patient or blood cells from different female donors. Values are expressed as fmol/million cells. The mean and SEM are shown. *, p<0.05, **, p<0.01, ***, p<0.001.

Following optimization of measuring TFV-DP in terms of cell numbers for each cell type, we evaluated TFV-DP in isolated cells (epithelial cells, fibroblasts, CD4^+^ T cells and CD14^+^ cells) from FRT tissues (EM, CX, ECX) which have been treated with TFV at 1 mg/ml for 24 hr and compared TFV-DP values to that seen in CD4^+^ T cells and monocyte-derived macrophages from blood. As seen in **Figure 2**, analysis of isolated cells from FRT tissues showed a significant variation in TFV-DP for the different cell types. TFV-DP in epithelial cells were 5-fold greater than in fibroblasts, with mean TFV-DP values of 2.5×10^6^ fmol/million cells versus 0.5×10^6^ fmol/million cells, respectively (p = 0.0006). Interestingly, TFV-DP in epithelial cells were approximately 50-fold and 125-fold greater than in FRT CD14^+^ cells and CD4^+^ T cells, respectively (p = 0.003 and 0.0002). Additionally, TFV-DP in FRT CD4^+^ T cells was comparable to that measured in blood CD4^+^ T cells. Our studies suggest that a cellular gradient in which individual FRT cell types are exposed to TFV results in TFV-DP concentrations that vary with cell type with epithelial cells > fibroblasts > CD14^+^ cells and CD4^+^ T cells.

### 3. TFV-DP in epithelial cells vary with the FRT tissue analyzed

**Figure 3:**
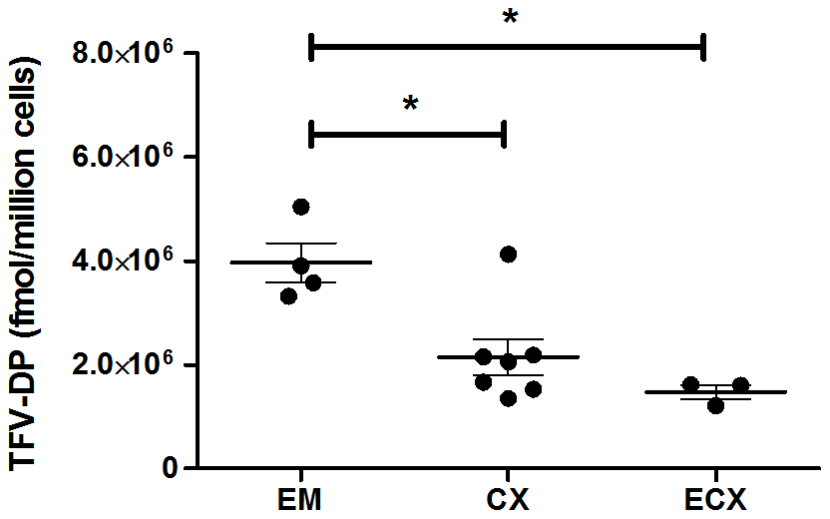
TFV-DP levels in epithelial cells FRT endometrium, endocervix and ectocervix tissue. TFV-DP levels were measured by LC-MS/MS in purified cultures of EM (n = 4), CX (n = 7) and ECX (n = 3) epithelial cells treated with TFV (1 mg/ml) for 24 hr. Each circle represents an individual patient. Values are expressed as fmol/million cells. The mean and SEM are shown. *, p<0.05.

To determine the variation of TFV-DP in individual cells from different FRT locations, purified epithelial cells from the upper and lower FRT were incubated with TFV at 1 mg/ml for 24 hr. As seen in **Figure 3**, TFV-DP in EM epithelial cells (4×10^6^ fmol/million cells) were 1.9-fold (2.1×10^6^ fmol/million cells) and 2.7-fold (1.5×10^6^ fmol/million cells) greater than in epithelial cells from CX or ECX, respectively (p = 0.04 and 0.05). In contrast, TFV-DP concentrations in fibroblasts from EM, CX and ECX were comparable in our preliminary results and did not vary with the site analyzed (data not shown).

### 4. Effect of estradiol and/or progesterone on TFV-DP in FRT epithelial cells

**Figure 4:**
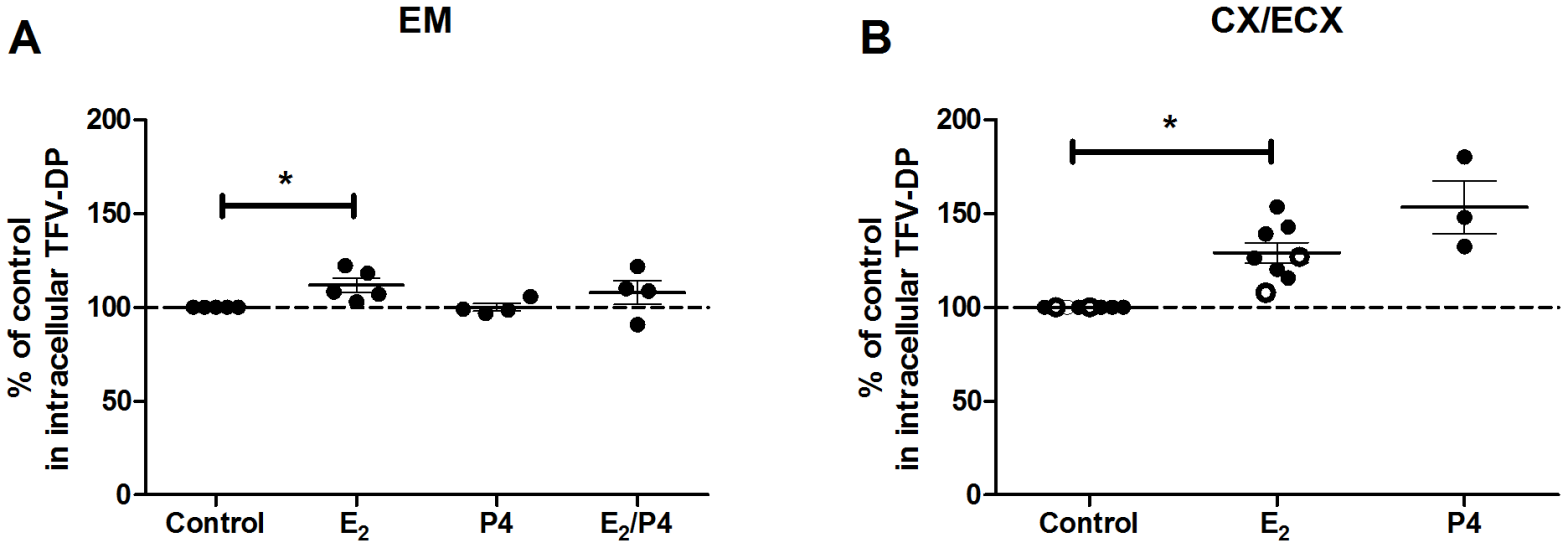
Effect of estradiol and/or progesterone on TFV-DP levels in epithelial cells from endometrium, endocervix and ectocervix. TFV-DP levels were measured by LC-MS/MS in polarized cultures of FRT epithelial cells treated with TFV (1 mg/ml) and estradiol (5×10^−8^ M), progesterone (1×10^−7^ M), either alone or the combination for 24 hr. Data were normalized to % of control values from (A) EM epithelial cells (n = 4–5) and (B) CX/ECX epithelial cells (n = 3–8). Dashed line indicates an assigned value of 100. Each circle represents a different patient. Dark circles indicate (A) EM epithelial cells and (B) CX epithelial cells. Open circles indicate ECX epithelial cells. The mean and SEM are shown. *, p<0.05.

To determine whether E_2_ and P4 alone or in combination have an effect on TFV-DP in primary FRT epithelial cells, purified cells were incubated with TFV (1 mg/ml) and E_2_ (5×10^−8^ M), P4 (1×10^−7^ M) or a combination of E_2_ and P4 for 24 hr. As shown with epithelial cells from 4-5 patients (**Figure 4A**), E_2_ treatment significantly increased TFV-DP in polarized EM epithelial cells. In contrast, P4 had no effect. Interestingly, however, when E_2_ was added along with P4, no stimulatory effect was observed. Similarly, we found that E_2_ treatment of CX and ECX epithelial cells grown on cell inserts increased TFV-DP from 6 patients (**Figure 4B**). In these studies, data from CX and ECX were combined owing to the limited numbers of tissues available for this study. Also shown in **Figure 4B** are studies carried out with P4.

### 5. Effect of estradiol and/or progesterone on TFV-DP in CD4^+^ T cells from the FRT and blood

**Figure 5:**
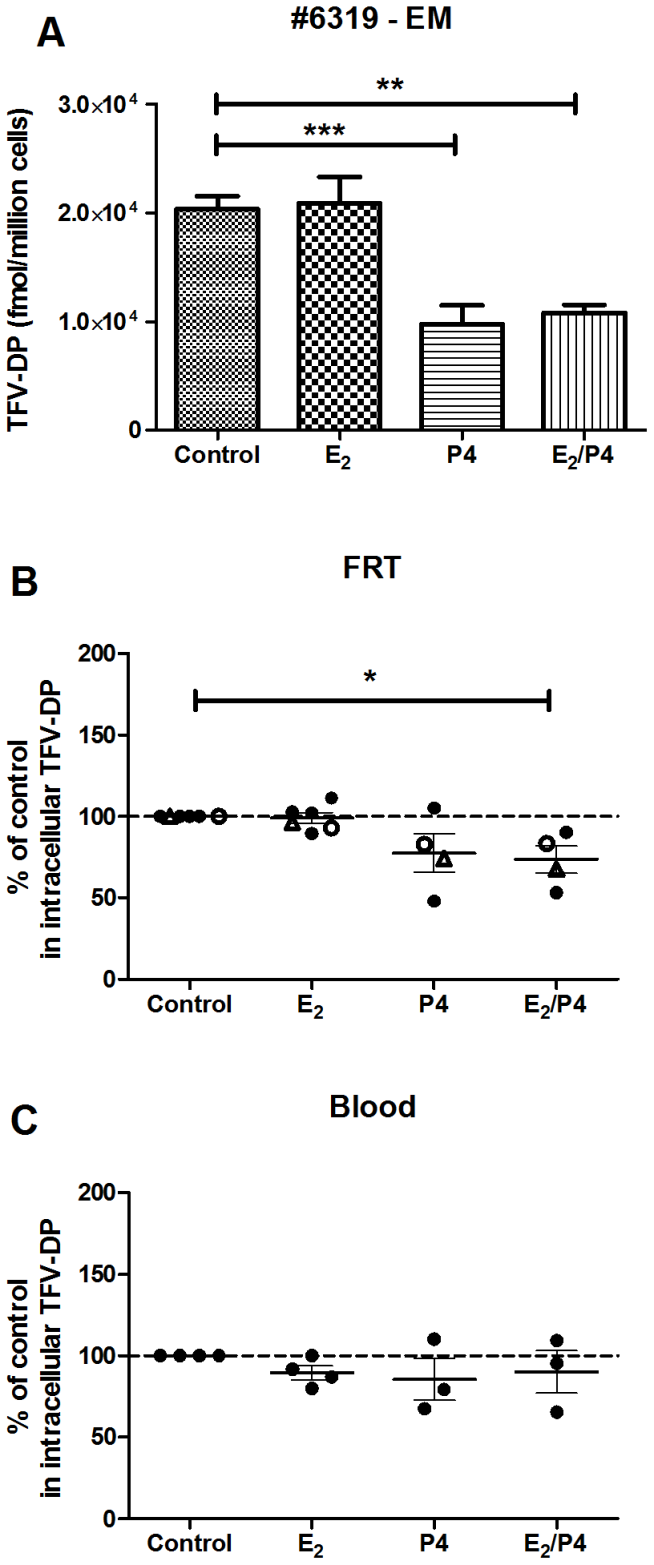
Effect of estradiol and/or progesterone on TFV-DP levels in CD4^+^ T cells from FRT and blood. TFV-DP levels were measured by LC-MS/MS in purified FRT and blood CD4^+^ T cells treated with TFV (1 mg/ml) and either estradiol (5×10^−8^ M), progesterone (1×10^−7^ M), or the combination for 24 hr. Values are expressed as fmol/million cells in (A) EM CD4^+^ T cells (patient number 6319). The Bars represent mean and SEM from triplicate cultures. Data were normalized to % of control values from (B) FRT CD4^+^ T cells (n = 4–6) and (C) blood CD4^+^ T cells (n = 3–4). Dashed line indicates an assigned value of 100. Each circle or triangle represents a different patient or blood donor. Dark circles indicate (B) EM CD4^+^ T cells or (C) blood CD4^+^ T cells. Open circles indicate CX CD4^+^ T cells and triangles indicate ECX CD4^+^ T cells. The mean and SEM are shown. *, p<0.05, **, p<0.01, ***, p<0.001.

To determine whether E_2_ and/or P4 influence TFV-DP concentrations in FRT and blood CD4^+^ T cells, purified CD4^+^ T cells were isolated by magnetic bead selection and incubated with TFV (1 mg/ml) using the same hormone protocol described above for epithelial cells and fibroblasts. As shown with FRT CD4^+^ T cells from a representative patient (**Figure 5A**) or 4–6 patients (**Figure 5B**), P4 in combination with E_2_ treatment significantly decreased TFV-DP concentrations. Moreover, P4 alone significantly decreased TFV-DP concentrations in 3/4 patients whereas E_2_ had no effect in 4/4 experiments. In contrast, as seen in **Figure 5C**, TFV-DP concentrations in blood CD4^+^ T cells from female donors were unaffected by E_2_ and/or P4.

### 6. Effect of estradiol and/or progesterone on TFV-DP in FRT fibroblasts

To determine whether E_2_ and/or P4 has an effect on TFV-DP in primary FRT fibroblasts, isolated fibroblasts were treated with TFV (1 mg/ml) and sex hormones. We found that E_2_ and P4 either alone or in combination had no effect on TFV-DP in FRT fibroblasts. Interestingly, P4 either alone or in combination with E_2_ treatment decreased TFV-DP 20–40% in fibroblasts from CX and ECX in 3 out of 5 experiments (data not shown). Further studies are needed to determine the significance of these preliminary results.

## Discussion

In the present study we demonstrate that immune and non-immune cells from the human FRT take up TFV and activate it to TFV-DP, the active form that inhibits reverse transcriptase to block HIV infection of target cells. To the best of our knowledge this study is the first to determine: i) the concentrations of TFV-DP in different cell types at different locations within the FRT and ii) the effect of sex hormones on these concentrations. We found that TFV-DP concentrations vary significantly with the cell type analyzed and the site within the FRT, suggesting the presence of a gradient of intracellular TFV-DP in which epithelial cells > fibroblasts > CD4^+^ T cells and CD14^+^ cells. The data presented also shows that E_2_ and P4 are directly involved in regulating intracellular TFV-DP concentrations in epithelial cells and CD4^+^ T cells, one of the main target cells of HIV.

A key observation from this study was that FRT epithelial cells and fibroblasts are able to phosphorylate TFV and generate intracellular TFV-DP more efficiently than CD4^+^ T cells and CD14^+^ cells. TFV-DP concentrations achieved in these non-target cells were considerably greater than in HIV target cells. As assessed per million cells, TFV-DP in FRT epithelial cells were approximately 53-fold and 125-fold greater than in FRT CD14^+^ cells and CD4^+^ T cells, respectively. TFV-DP in FRT fibroblasts were approximately 10-fold and 25-fold greater than in FRT CD14^+^ cells and CD4^+^ T cells, respectively. Interestingly, concentrations of TFV-DP in FRT CD4^+^ T cells were comparable to that measured in blood CD4^+^ T cells. The FRT mucosa is composed of multiple cell types and the predominant cell types are epithelial cells and fibroblasts [24]. Our studies suggest that the predominance of these epithelial cells with their high intracellular concentrations of TFV-DP may lead to an over estimation of TFV-DP concentrations ascribed to HIV target cells as reported tissue biopsy or cytobrush samples [12].

The TFV-DP concentration measured in this study describe a potential gradient that occurs both with the cell type (epithelial cells > fibroblasts > CD14^+^ cells and CD4^+^ T cells) and FRT location (epithelial cells, EM > CX or ECX). The relatively low TFV-DP in CD4^+^ T cells and macrophages suggest that, because of sequestration of TFV by epithelial cells and fibroblasts accounts for 50-70% of non-myometrial cells in the FRT, some ART drugs may not achieve sufficient concentrations to effectively suppress local replication of HIV in HIV target cells [25]. Based on preliminary studies from our laboratory (Bodwell et al, Unpublished observation), we know that epithelial and stromal fibroblasts possess the enzymatic machinery necessary to both activate TFV and inactivate TFV-DP. Once inactivated, TFV can readily move out of these cells to be available for uptake and metabolic activation by HIV target cells [14]. Our results in the present study suggest that epithelial cells and fibroblasts are a repository of TFV-DP. At the heart of this hypothesis is the recognition that epithelial cells and fibroblasts might act either as a sink to decrease TFV availability to CD4^+^ T cells and macrophages in the FRT, or on conversion of TFV-DP to TFV, increase TFV available to HIV target cells.

Given the high intracellular active tenofovir levels achieved in these studies, it is possible that FRT epithelial cells may be prone to toxic side effects. Tenofovir as a DNA chain terminator NRTI drug causes mitochondrial dysfunction in renal proximal tubules [26]–[28]. MTN-001 findings showed that TFV-PP levels in biopsy vagina tissues are ∼130 times higher with vaginal gel TFV compared to that measured in the vagina after oral drug administration [12]. The data presented here implies that active drug levels, specifically in genital epithelial cells, could reach substantially higher peaks *in situ*.

Recognizing that sex hormones affect most if not all metabolic events in the FRT [29], we asked whether E_2_ and/or P4 influence the intracellular concentrations of TFV-DP within immune and non-immune cells in the FRT. Our findings indicate that the effects of sex hormones on TFV-DP are quite divergent and dependent on the cell type. Thus while E_2_ alone in culture increased TFV-DP in EM and CX/ECX epithelial cells, P4 in combination with E_2_ decreased TFV-DP in CD4^+^ T cells from FRT tissues. An unexpected finding in our study was that whereas both epithelial cells and fibroblasts contain E_2_ and P4 receptors and are responsive to sex hormones [29], hormone treatment of fibroblasts failed to alter TDV-DP in fibroblasts. Just why these cells differ in their responsiveness to E_2_ remains unclear. In previous studies we have seen differential responses between fibroblasts from different sites in the FRT. For example, whereas E_2_ stimulates hepatocyte growth factor (HGF) secretion by EM fibroblasts, it had no effect on CX and ECX fibroblast secretion of HGF [30]. In response to E_2_, EM epithelial cell secretion of HBD_2_ and Elafin increased, but was inhibited when vaginal epithelial cells were treated with E_2_ [31]. Our finding that fibroblasts are non-responsive to hormones with respect to TFV-DP concentrations suggests the possibility that the two subtypes of estrogen receptor (ER), ERα to ERβ in fibroblasts might be different from that in epithelial cells. Since E_2_ exerts its molecular actions by interactions with ERα and ERβ, which are now recognized as consisting of multiple isoforms [32], further studies are needed to identify the unique mechanisms involved in TFV-DP availability.

We previously hypothesized that women are most susceptible to HIV infection when E_2_ and P4 levels are highest during ovulation and the secretory phase of the menstrual cycle [5]. Protection in the FRT is shared between the innate and adaptive immune systems and both are under hormonal control [6], [7]. Given this immune regulation by sex hormones, we asked whether E_2_ and P4 influence the intracellular concentrations of TFV-DP in cells from the FRT. Our findings that E_2_ increases TFV-DP in epithelial cells from the EM and CX/ECX suggest that when E_2_ levels are elevated, intracellular TFV-DP in epithelial cells will be high. If so, then increased uptake and activation to TFV-DP by epithelial cells might lower TFV available for uptake by HIV target cells. Conversely, when E_2_ levels are low, as during the proliferative phase of the menstrual cycle, epithelial cell conversion of TFV-DP back to TFV could contribute to enhanced protection, measured as a greater availability of TFV for HIV target cells. Further studies are needed to determine whether epithelial cells provide a meaningful source of TFV for HIV target cells or if hormonal changes compromise microbicide mediated protection against HIV in the FRT.

Previously, we demonstrated that E_2_ enhances nucleotidase (NT) expression and biological activity in epithelial cells and fibroblasts from upper and lower FRT [20]. E_2_, but not P4 either alone or in combination, increased gene expression of Cytosolic 5′-nucleotidase after 2 or 4 hr in EM epithelial cells but not epithelial cells or fibroblasts from other sites. However, in studies using a modified 5′-NT biological assay for nucleotidases, E_2_ increased NT activity in epithelial cells and fibroblasts from the EM, CX and ECX at 24 and 48 hr. Nucleotidases have phosphatase activity and are involved in the catabolism of nucleotides through dephosphorylation of nucleotide terminal phosphates with a preference for nucleotide monophosphates [33] and have been implicated in TFV metabolism. Our findings of increased intracellular TFV-DP in epithelial cells in response to E_2_ are opposite to our earlier findings of enhanced NT activity, since enhanced NT activity would be expected to decrease TFV-DP, not enhance them as found. The present studies indicate that either NT enzymes are not key players in the catabolism of TFV-DP or that E_2_ enhances formation through the stimulation of kinase activity to offset changes in increased phosphatase activity. Future studies are needed that focus on the dynamic balance of the enzymes (kinases) necessary for formation of activated microbicide TFV-DP and those required for TFV-DP degradation (nucleotidases and phosphatases) in HIV-target cells.

Beyond the E_2_ effects on TFV-DP, we found that P4 in combination with E_2_ decreases TFV-DP in CD4^+^ T cells from the FRT. In studies to measure TFV and TFV-DP concentrations in blood and blood PBMC, Coleman et al. found that the use of hormonal contraception (oral and injectable) was associated with decreased serum and intracellular PBMC TFV concentrations [34]. Since as many as 70% of women in TFV PrEP trials used some form of chemical contraception, our findings that sex hormones alter TFV-DP in immune and non-immune cells from the FRT, when considered along with those of Coleman et al., provide compelling evidence that studies are needed to determine the extent to which intracellular TFV-DP within CD4^+^ T cells and macrophages in the FRT change with stage of the menstrual cycle and hormonal contraceptive use and whether these changes enhance or reduce susceptibility to HIV infection.

In conclusion, these studies demonstrate measurable TFV-DP in epithelial cells, fibroblasts and immune cells from the upper and lower FRT. Intracellular concentrations vary with the cell type and location within the FRT. In addition, these results indicate that sex hormones regulate the production of TFV-DP in epithelial cells, suggesting that they may alter both TFV availability to HIV target cells and protection against HIV in the FRT. Future studies are needed to understand the mechanisms involved in the modulation of microbicide concentrations by sex hormones and chemical contraceptives in FRT tissues and how these relate to PrEP trial outcomes in order to more fully define the complex interactions of the endocrine system and its influence on microbicide efficacy and protection against HIV.

## References

[1] UNAIDS (2013) UNAIDS report on the global AIDS.

[2] CAC (2003) HIV/AIDS Surveillance Report.

[3] Ghosh M, Rodriguez-Garcia M, Wira CR (2013) Immunobiology of genital tract trauma: endocrine regulation of HIV acquisition in women following sexual assault or genital tract mutilation. Am J Reprod Immunol 69 Suppl 1: 51–60. 23034063 10.1111/aji.12027 PMC3566368

[4] (2012) HIV/AIDS.

[5] Wira CR, Fahey JV (2008) A new strategy to understand how HIV infects women: identification of a window of vulnerability during the menstrual cycle. AIDS 22: 1909–1917. 18784454 10.1097/QAD.0b013e3283060ea4 PMC2647143

[6] Hickey DK, Patel MV, Fahey JV, Wira CR (2011) Innate and adaptive immunity at mucosal surfaces of the female reproductive tract: stratification and integration of immune protection against the transmission of sexually transmitted infections. J Reprod Immunol 88: 185–194. 21353708 10.1016/j.jri.2011.01.005 PMC3094911

[7] Wira CR, Fahey JV, Sentman CL, Pioli PA, Shen L (2005) Innate and adaptive immunity in female genital tract: cellular responses and interactions. Immunol Rev 206: 306–335. 16048557 10.1111/j.0105-2896.2005.00287.x

[8] Heneine W, Kashuba A (2012) HIV Prevention by Oral Preexposure Prophylaxis. Cold Spring Harb Perspect Med 2: a007419. 22393535 10.1101/cshperspect.a007419 PMC3282498

[9] Rohan LC, Moncla BJ, Kunjara Na Ayudhya RP, Cost M, Huang Y, et al (2010) In vitro and ex vivo testing of tenofovir shows it is effective as an HIV-1 microbicide. PLoS One 5: e9310. 20174579 10.1371/journal.pone.0009310 PMC2824823

[10] van der Straten A, Van Damme L, Haberer JE, Bangsberg DR (2012) Unraveling the divergent results of pre-exposure prophylaxis trials for HIV prevention. AIDS 26: F13–19. 22333749 10.1097/QAD.0b013e3283522272

[11] Romano J, Kashuba A, Becker S, Cummins J, Turpin J, et al (2013) Pharmacokinetics and Pharmacodynamics in HIV Prevention; Current Status and Future Directions: A Summary of the DAIDS and BMGF Sponsored Think Tank on Pharmacokinetics (PK)/Pharmacodynamics (PD) in HIV Prevention. AIDS Res Hum Retroviruses 29: 1418–1427. 23614610 10.1089/aid.2013.0122 PMC3809377

[12] Hendrix CW, Chen BA, Guddera V, Hoesley C, Justman J, et al (2013) MTN-001: randomized pharmacokinetic cross-over study comparing tenofovir vaginal gel and oral tablets in vaginal tissue and other compartments. PLoS One 8: e55013. 23383037 10.1371/journal.pone.0055013 PMC3559346

[13] Chen J, Flexner C, Liberman RG, Skipper PL, Louissaint NA, et al (2012) Biphasic elimination of tenofovir diphosphate and nonlinear pharmacokinetics of zidovudine triphosphate in a microdosing study. J Acquir Immune Defic Syndr 61: 593–599. 23187888 10.1097/QAI.0b013e3182717c98 PMC3509498

[14] Anderson PL, Kiser JJ, Gardner EM, Rower JE, Meditz A, et al (2011) Pharmacological considerations for tenofovir and emtricitabine to prevent HIV infection. J Antimicrob Chemother 66: 240–250. 21118913 10.1093/jac/dkq447 PMC3019086

[15] Fahey JV, Schaefer TM, Channon JY, Wira CR (2005) Secretion of cytokines and chemokines by polarized human epithelial cells from the female reproductive tract. Hum Reprod 20: 1439–1446. 15734755 10.1093/humrep/deh806

[16] Fahey JV, Wira CR (2002) Effect of menstrual status on anti-bacterial activity and secretory leukocyte protease inhibitor production by human uterine epithelial cells in culture. J Infect Dis 185: 1606–1613. 12023766 10.1086/340512

[17] Meter RA, Wira CR, Fahey JV (2005) Secretion of monocyte chemotactic protein-1 by human uterine epithelium directs monocyte migration in culture. Fertil Steril 84: 191–201. 16009177 10.1016/j.fertnstert.2005.01.104

[18] Koumas L, King AE, Critchley HO, Kelly RW, Phipps RP (2001) Fibroblast heterogeneity: existence of functionally distinct Thy 1(+) and Thy 1(-) human female reproductive tract fibroblasts. Am J Pathol 159: 925–935. 11549585 10.1016/S0002-9440(10)61768-3 PMC1850439

[19] MacDonald EM, Savoy A, Gillgrass A, Fernandez S, Smieja M, et al (2007) Susceptibility of human female primary genital epithelial cells to herpes simplex virus, type-2 and the effect of TLR3 ligand and sex hormones on infection. Biol Reprod 77: 1049–1059. 17881767 10.1095/biolreprod.107.063933

[20] Shen Z, Fahey JV, Bodwell JE, Rodriguez-Garcia M, Rossoll RM, et al (2013) Estradiol regulation of nucleotidases in female reproductive tract epithelial cells and fibroblasts. PLoS One 8: e69854. 23936114 10.1371/journal.pone.0069854 PMC3723851

[21] Rodriguez-Garcia M, Biswas N, Patel MV, Barr FD, Crist SG, et al (2013) Estradiol reduces susceptibility of CD4+ T cells and macrophages to HIV-infection. PLoS One 8: e62069. 23614015 10.1371/journal.pone.0062069 PMC3629151

[22] McNatty KP, Baird DT, Bolton A, Chambers P, Corker CS, et al (1976) Concentration of oestrogens and androgens in human ovarian venous plasma and follicular fluid throughout the menstrual cycle. J Endocrinol 71: 77–85. 978120 10.1677/joe.0.0710077

[23] Patterson KB, Prince HA, Kraft E, Jenkins AJ, Shaheen NJ, et al (2011) Penetration of tenofovir and emtricitabine in mucosal tissues: implications for prevention of HIV-1 transmission. Sci Transl Med 3: 112re114. 10.1126/scitranslmed.3003174 PMC3483088 22158861

[24] Givan AL, White HD, Stern JE, Colby E, Gosselin EJ, et al (1997) Flow cytometric analysis of leukocytes in the human female reproductive tract: Comparison of Fallopian tube, uterus, cervix, and vagina. Am J Reprod Immunol 38: 350–359. 9352027 10.1111/j.1600-0897.1997.tb00311.x

[25] Kashuba AD, Patterson KB, Dumond JB, Cohen MS (2012) Pre-exposure prophylaxis for HIV prevention: how to predict success. Lancet 379: 2409–2411. 22153566 10.1016/S0140-6736(11)61852-7 PMC3652584

[26] Kohler JJ, Hosseini SH, Hoying-Brandt A, Green E, Johnson DM, et al (2009) Tenofovir renal toxicity targets mitochondria of renal proximal tubules. Lab Invest 89: 513–519. 19274046 10.1038/labinvest.2009.14 PMC2674517

[27] Kohler JJ, Hosseini SH (2011) Subcellular renal proximal tubular mitochondrial toxicity with tenofovir treatment. Methods Mol Biol 755: 267–277. 21761311 10.1007/978-1-61779-163-5_22

[28] Abraham P, Ramamoorthy H, Isaac B (2013) Depletion of the cellular antioxidant system contributes to tenofovir disoproxil fumarate - induced mitochondrial damage and increased oxido-nitrosative stress in the kidney. J Biomed Sci 20: 61. 23957306 10.1186/1423-0127-20-61 PMC3765371

[29] Wira CR, Fahey JV, Ghosh M, Patel MV, Hickey DK, et al (2010) Sex hormone regulation of innate immunity in the female reproductive tract: the role of epithelial cells in balancing reproductive potential with protection against sexually transmitted pathogens. Am J Reprod Immunol 63: 544–565. 20367623 10.1111/j.1600-0897.2010.00842.x PMC3837356

[30] Coleman KD, Wright JA, Ghosh M, Wira CR, Fahey JV (2009) Estradiol modulation of hepatocyte growth factor by stromal fibroblasts in the female reproductive tract. Fertil Steril 92: 1107–1109. 19423096 10.1016/j.fertnstert.2008.10.047 PMC4322385

[31] Patel MV, Fahey JV, Rossoll RM, Wira CR (2013) Innate immunity in the vagina (part I): estradiol inhibits HBD2 and elafin secretion by human vaginal epithelial cells. Am J Reprod Immunol 69: 463–474. 23398087 10.1111/aji.12078 PMC3837349

[32] Murphy AJ, Guyre PM, Wira CR, Pioli PA (2009) Estradiol regulates expression of estrogen receptor ERalpha46 in human macrophages. PLoS One 4: e5539. 19440537 10.1371/journal.pone.0005539 PMC2678254

[33] Hunsucker SA, Mitchell BS, Spychala J (2005) The 5'-nucleotidases as regulators of nucleotide and drug metabolism. Pharmacol Ther 107: 1–30. 15963349 10.1016/j.pharmthera.2005.01.003

[34] Coleman J, Chaturvedula A, Heendrix C, Team M-P (2012) Method of hormonal contraception is associated with lower tenofovir concentration in healthy women (MTN-001): implications for pre-exposure prophylaxis. XIX International AIDS Conference AIDS 2012. Washington, DC.

